# Tris[hexa­amminecobalt(III)] bis­[tri­oxalato­cobaltate(II)] chloride dodeca­hydrate

**DOI:** 10.1107/S1600536812026414

**Published:** 2012-06-16

**Authors:** Ruijng Tian, Yan Yan, Cailing Zhang, Liyan Wang, Qinhe Pan

**Affiliations:** a Department of Materials and Chemical Engineering, Ministry of Education Key Laboratory of Advanced Materials of Tropical Island Resources, Hainan University, Haikou 570228, People’s Republic of China; b State Key Laboratory of Inorganic Synthesis and Preparative Chemistry, College of Chemistry, Jilin University, Changchun 130012, People’s Republic of China

## Abstract

The title compound, [Co^III^(NH_3_)_6_]_3_[Co^II^(C_2_O_4_)_3_]_2_Cl·12H_2_O, was synthesized under hydro­thermal conditions. The asymmetric unit comprises two [Co(NH_3_)_6_]^3+^ cations, one located on a threefold axis and the other on a site of symmetry -3, a [Co(C_2_O_4_)_3_]^4+^ anion, located on a threefold axis, one sixth of a chloride anion [disordered over two sites, one threefold (site occupancy = 0.5) and the other -3 (site occupancy (0.25)] and two water molecules. Both Co^III^ centers are six-coordinated by NH_3_ mol­ecules, forming [Co(NH_3_)_6_]^3+^ octa­hedra, with Co—N distances in the range 1.958 (2)–1.977 (3) Å. The title structure gives the first example of the [Co(C_2_O_4_)_3_]^4−^ anion, with the distorted octa­hedral environment of Co^II^ center formed by six O atoms from three oxalate residues. The Co—O bond lengths are 2.0817 (18) to 2.0979 (18) Å. Multiple N—H⋯O, N—H⋯Cl and O—H⋯O hydrogen bonds link the cations, anions and water mol­ecules into a three-dimensional network.

## Related literature

For metal phosphates and germanates templated by metal complexes, see: Wang *et al.* (2003 ▶, 2006 ▶); Pan *et al.* (2005 ▶, 2008 ▶). For our continued research inter­est, see: Pan *et al.* (2010**a* ▶,b* ▶, 2011 ▶). For a compound containing the [Co^III^(NH_3_)_6_]^3+^ cation, see: Wu *et al.* (2012 ▶).
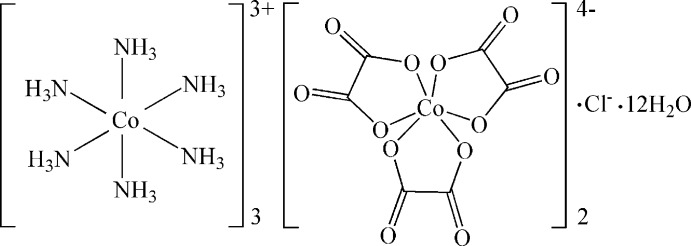


## Experimental

### 

#### Crystal data

[Co(NH_3_)_6_][Co(C_2_O_4_)_3_]Cl·12H_2_O
*M* *_r_* = 1381.02
Trigonal, 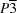

*a* = 12.2138 (4) Å
*c* = 9.9090 (8) Å
*V* = 1280.15 (12) Å^3^
*Z* = 1
Mo *K*α radiation
μ = 1.75 mm^−1^
*T* = 296 K
0.30 × 0.15 × 0.15 mm

#### Data collection

Bruker APEXII CCD area-detector diffractometer
Absorption correction: multi-scan (*SADABS*; Bruker, 2005 ▶) *T*_min_ = 0.737, *T*_max_ = 0.769
7736 measured reflections
1923 independent reflections
1514 reflections with *I* > 2σ(*I*)
*R*_int_ = 0.030

#### Refinement

*R*[*F* ^2^ > 2σ(*F* ^2^)] = 0.036
*wR*(*F* ^2^) = 0.108
*S* = 1.16
1923 reflections
115 parameters
1 restraint
H-atom parameters constrained
Δρ_max_ = 0.61 e Å^−3^
Δρ_min_ = −1.09 e Å^−3^

### 

Data collection: *APEX2* (Bruker, 2005 ▶); cell refinement: *SAINT* (Bruker, 2005 ▶); data reduction: *SAINT* program(s) used to solve structure: *SHELXS97* (Sheldrick, 2008 ▶); program(s) used to refine structure: *SHELXL97* (Sheldrick, 2008 ▶); molecular graphics: *SHELXTL* (Sheldrick, 2008 ▶); software used to prepare material for publication: *SHELXTL*.

## Supplementary Material

Crystal structure: contains datablock(s) I, global. DOI: 10.1107/S1600536812026414/yk2060sup1.cif

Structure factors: contains datablock(s) I. DOI: 10.1107/S1600536812026414/yk2060Isup2.hkl

Additional supplementary materials:  crystallographic information; 3D view; checkCIF report

## Figures and Tables

**Table 1:** Hydrogen-bond geometry (Å, °)

*D*—H⋯*A*	*D*—H	H⋯*A*	*D*⋯*A*	*D*—H⋯*A*
N1—H1*A*⋯Cl1′	0.89	2.62	3.176 (15)	121
N1—H1*A*⋯O1^i^	0.89	2.27	3.055 (4)	147
N1—H1*B*⋯O2^ii^	0.89	2.40	2.950 (4)	120
N1—H1*B*⋯O2^iii^	0.89	2.52	3.151 (4)	128
N2—H2⋯O4^iv^	0.91	2.09	2.993 (3)	171
N2—H2*A*⋯O1*W*	0.91	2.18	3.047 (4)	160
N2—H2*B*⋯O2*W*	0.91	2.15	2.958 (3)	147
N3—H3⋯O3^v^	0.91	2.09	2.988 (3)	172
N3—H3*A*⋯O2*W*	0.91	2.19	3.051 (3)	157
N3—H3*B*⋯O1*W*	0.91	2.36	3.120 (4)	141
O1*W*—H1*W*⋯O1	0.87	2.13	2.971 (4)	162
O1*W*—H1*WA*⋯O1^vi^	0.87	2.32	2.973 (4)	132
O2*W*—H2*W*⋯O2^vii^	0.87	1.97	2.830 (3)	171
O2*W*—H2*WA*⋯O4^viii^	0.87	2.06	2.868 (3)	154

Symmetry codes: (i) 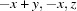; (ii) 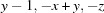; (iii) 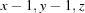; (iv) 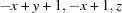; (v) 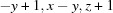; (vi) 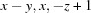; (vii) 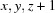; (viii) 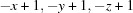.

**Fig. 1.**
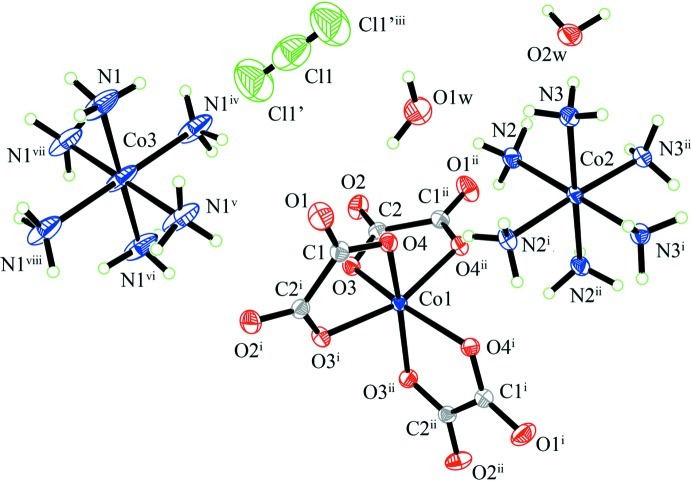
A view of the asymmetric unit of title compound. Ellipsoids are drawn at the 50% probability level. [Symmetry codes: (i) 1-y, x-y, z; (ii) 1-x+y, 1 - x, z; (iii) -x, -y, 1 - z; (iv) -x + y, -x, z; (v) -y, x-y, z; (vi) -x, -y, -z; (vii) y, -x + y, -z; (viii) x-y, x, -z.

## supplementary crystallographic information

### Comment

Recently, more attention has been paid to employ transition metal complexes as templates for supramolecular structures, because they are versatile and can be made with a wide variety of shapes and charges. Up to now, transition metal cTris[hexaamminecobalt(III)] bis[trioxalatocobaltate(II)] chloride dodecahydrateomplexes have been introduced into the synthesis of various open-framework materials, including metal phosphates (Wang *et al.*, 2003,2006), germanates (Pan *et al.*, 2005,2008). Our continued interest has been focused on the synthesis of microporous open-framework metal-organic structures by introducing transition metal complexes as templates (Pan *et al.*, 2010**a*,b*,2011). Unexpectedly, in the reaction of Co(OAc)_2_.4H_2_O, Co(NH_3_)_6_Cl_3_, and K_2_C_2_O_4_ the title compound, [Co^III^(NH_3_)_6_]_3_[Co^II^(C_2_O_4_)_3_]_2_.Cl.12H_2_O, was obtained.

The title compound is composed of [Co(NH_3_)_6_]^3+^ cations, the counterions [Co(C_2_O_4_)_3_]^4-^ and Cl^-^ anions, and two water molecules, as shown in Figure 1. The crystal structure contains two Co^III^ centers, one is located on a threefold rotation axis, and the other is located at a 3 position. Each Co^III^ center is six-coordinated by NH_3_ molecules to form octahedral [Co(NH_3_)_6_]^3+^ cations, similar to that observed in [Co(NH_3_)_6_]_2_(NO_3_)Cl_5_ (Wu *et al.*, 2012). The Co—N distances are in the range 1.958 (2)–1.977 (3) Å. The crystal structure also contains one Co^II^ center, which is located on a threefold rotation axis. It is coordinted by six O atoms from three different oxalate residues to form [Co(C_2_O_4_)_3_]^4-^ anion having a slightly distorted octahedral geometry, with the distances Co—O ranging from 2.0804 (17) to 2.0968 (17) Å. The Cl^-^ anion is splitted into two positions, Cl1 and Cl1'. The [Co(III)(NH_3_)_6_]^3+^ cations, [Co(II)(C_2_O_4_)_3_]^4-^ anions, Cl^-^ anions, and water molecules form an extensive hydrogen-bonding network, with the distance of N—H···N hydrogen bonds of 2.751 (4) Å, the distance of N—H···Cl hydrogen bonds of 3.169 (9) Å, the distance of N—H···O hydrogen bonds in the range of 2.959 (3)–3.147 (3) Å, and the distance of O—H···O hydrogen bonds in the range of 2.829 (3)–2.973 (4) Å (Table 1).

### Experimental

In a typical synthesis, a mixture of Co(OAc)_2_.4H_2_O (0.250 g), Co(NH_3_)_6_Cl_3_ (0.1 g), K_2_C_2_O_4_ (0.276 g), and H_2_O (5 ml), was placed into a 20 ml Teflon-lined reactor and heated to 100 °C for 3 days under autogenous pressure. Orange rod-like crystals were obtained.

### Refinement

All H atoms were positioned geometrically (N—H = 0.91 Å, O—H = 0.87 Å) and allowed to ride on their parent atoms, with *U*_iso_(H) = 1.2*U*_eq_(parent atom).

### Crystal data

**Table:**

[Co(NH_3_)_6_][Co(C_2_O_4_)_3_]Cl·12H_2_O	*D*_x_ = 1.791 Mg m^−^^3^
*M**_r_* = 1381.02	Mo *K*α radiation, λ = 0.71073 Å
Trigonal, *P*3	Cell parameters from 7736 reflections
Hall symbol: -P 3	θ = 2.1–27.2°
*a* = 12.2138 (4) Å	µ = 1.75 mm^−^^1^
*c* = 9.9090 (8) Å	*T* = 296 K
*V* = 1280.15 (12) Å^3^	Rod, orange
*Z* = 1	0.30 × 0.15 × 0.15 mm
*F*(000) = 716	

### Data collection

**Table:**

Bruker APEXII CCD area-detector diffractometer	1923 independent reflections
Radiation source: fine-focus sealed tube	1514 reflections with *I* > 2σ(*I*)
Graphite monochromator	*R*_int_ = 0.030
Detector resolution: 5.00 pixels mm^-1^	θ_max_ = 27.2°, θ_min_ = 2.1°
φ and ω scans	*h* = −8→15
Absorption correction: multi-scan (*SADABS*; Bruker, 2005)	*k* = −15→14
*T*_min_ = 0.737, *T*_max_ = 0.769	*l* = −12→12
7736 measured reflections	

### Refinement

**Table:**

Refinement on *F*^2^	Primary atom site location: structure-invariant direct methods
Least-squares matrix: full	Secondary atom site location: difference Fourier map
*R*[*F*^2^ > 2σ(*F*^2^)] = 0.036	Hydrogen site location: inferred from neighbouring sites
*wR*(*F*^2^) = 0.108	H-atom parameters constrained
*S* = 1.16	*w* = 1/[σ^2^(*F*_o_^2^) + (0.0482*P*)^2^ + 0.6892*P*] where *P* = (*F*_o_^2^ + 2*F*_c_^2^)/3
1923 reflections	(Δ/σ)_max_ = 0.011
115 parameters	Δρ_max_ = 0.61 e Å^−^^3^
1 restraint	Δρ_min_ = −1.09 e Å^−^^3^

### Special details

**Table:**

Geometry. All s.u.'s (except the s.u. in the dihedral angle between two l.s. planes) are estimated using the full covariance matrix. The cell s.u.'s are taken into account individually in the estimation of s.u.'s in distances, angles and torsion angles; correlations between s.u.'s in cell parameters are only used when they are defined by crystal symmetry. An approximate (isotropic) treatment of cell s.u.'s is used for estimating s.u.'s involving l.s. planes.
Refinement. Refinement of *F*^2^ against ALL reflections. The weighted *R*-factor *wR* and goodness of fit *S* are based on *F*^2^, conventional *R*-factors *R* are based on *F*, with *F* set to zero for negative *F*^2^. The threshold expression of *F*^2^ > σ(*F*^2^) is used only for calculating *R*-factors(gt) *etc*. and is not relevant to the choice of reflections for refinement. *R*-factors based on *F*^2^ are statistically about twice as large as those based on *F*, and *R*- factors based on ALL data will be even larger.

### Fractional atomic coordinates and isotropic or equivalent isotropic displacement parameters (Å^2^)

**Table:**

	*x*	*y*	*z*	*U*_iso_*/*U*_eq_	Occ. (<1)
Co1	0.6667	0.3333	0.14192 (6)	0.02287 (18)	
Co2	0.6667	0.3333	0.64732 (5)	0.02393 (18)	
Co3	0.0000	0.0000	0.0000	0.0646 (5)	
Cl1	0.0000	0.0000	0.5000	0.085 (5)	0.498 (19)
Cl1'	0.0000	0.0000	0.3949 (17)	0.118 (4)	0.251 (9)
O1	0.3041 (2)	0.0971 (2)	0.2657 (2)	0.0530 (6)	
O2	0.6796 (2)	0.65788 (19)	0.0164 (2)	0.0454 (6)	
O3	0.64285 (18)	0.45991 (18)	0.02214 (18)	0.0311 (4)	
O4	0.50524 (17)	0.24996 (18)	0.26488 (18)	0.0291 (4)	
N1	−0.1492 (2)	−0.0618 (3)	0.1172 (4)	0.0725 (11)	
H1A	−0.1436	−0.1075	0.1840	0.087*	
H1B	−0.2187	−0.1096	0.0692	0.087*	
H1C	−0.1528	0.0037	0.1514	0.087*	
N2	0.6170 (2)	0.4327 (2)	0.5344 (2)	0.0328 (5)	
H2B	0.6232	0.4999	0.5808	0.039*	
H2A	0.5333	0.3879	0.5132	0.039*	
H2	0.6626	0.4599	0.4568	0.039*	
N3	0.5188 (2)	0.2924 (2)	0.7604 (2)	0.0307 (5)	
H3B	0.4436	0.2449	0.7175	0.037*	
H3A	0.5208	0.3659	0.7815	0.037*	
H3	0.5185	0.2514	0.8372	0.037*	
C1	0.4097 (3)	0.1551 (3)	0.2134 (3)	0.0310 (6)	
C2	0.6835 (2)	0.5688 (2)	0.0718 (3)	0.0288 (6)	
O1W	0.3354 (2)	0.2406 (3)	0.5187 (3)	0.0747 (9)	
H1WA	0.2644	0.2152	0.5612	0.090*	
H1W	0.3234	0.1853	0.4567	0.090*	
O2W	0.5721 (2)	0.5645 (2)	0.7595 (2)	0.0523 (6)	
H2WA	0.5434	0.6113	0.7265	0.063*	
H2W	0.5970	0.5901	0.8416	0.063*	

### Atomic displacement parameters (Å^2^)

**Table:**

	*U*^11^	*U*^22^	*U*^33^	*U*^12^	*U*^13^	*U*^23^
Co1	0.0244 (2)	0.0244 (2)	0.0198 (3)	0.01220 (11)	0.000	0.000
Co2	0.0275 (2)	0.0275 (2)	0.0167 (3)	0.01377 (12)	0.000	0.000
Co3	0.0176 (3)	0.0176 (3)	0.1585 (15)	0.00881 (16)	0.000	0.000
Cl1	0.062 (2)	0.062 (2)	0.131 (13)	0.0310 (12)	0.000	0.000
Cl1'	0.148 (7)	0.148 (7)	0.060 (8)	0.074 (4)	0.000	0.000
O1	0.0294 (11)	0.0621 (15)	0.0463 (13)	0.0069 (10)	0.0097 (10)	−0.0024 (11)
O2	0.0507 (13)	0.0307 (11)	0.0548 (14)	0.0203 (10)	−0.0105 (11)	0.0070 (9)
O3	0.0394 (11)	0.0309 (10)	0.0263 (10)	0.0201 (9)	−0.0026 (8)	0.0002 (8)
O4	0.0269 (9)	0.0335 (10)	0.0246 (9)	0.0133 (8)	0.0009 (7)	−0.0008 (7)
N1	0.0262 (14)	0.0270 (14)	0.164 (4)	0.0127 (12)	0.0017 (18)	0.0026 (18)
N2	0.0411 (13)	0.0394 (13)	0.0220 (11)	0.0232 (11)	0.0011 (10)	0.0031 (9)
N3	0.0322 (12)	0.0354 (12)	0.0251 (11)	0.0172 (10)	0.0016 (9)	0.0010 (9)
C1	0.0286 (14)	0.0331 (14)	0.0308 (15)	0.0150 (12)	0.0000 (11)	0.0026 (11)
C2	0.0260 (13)	0.0287 (13)	0.0307 (14)	0.0129 (11)	0.0032 (11)	0.0031 (11)
O1W	0.0478 (15)	0.107 (2)	0.0662 (18)	0.0367 (16)	−0.0013 (13)	−0.0213 (17)
O2W	0.0805 (18)	0.0623 (15)	0.0407 (13)	0.0555 (15)	−0.0056 (12)	−0.0047 (11)

### Geometric parameters (Å, º)

**Table:**

Co1—O3^i^	2.0817 (18)	Cl1'—Cl1'^viii^	2.08 (3)
Co1—O3^ii^	2.0817 (18)	O1—C1	1.233 (3)
Co1—O3	2.0817 (18)	O2—C2	1.240 (3)
Co1—O4^ii^	2.0979 (18)	O3—C2	1.264 (3)
Co1—O4^i^	2.0979 (18)	O4—C1	1.270 (3)
Co1—O4	2.0979 (18)	N1—H1A	0.8900
Co2—N2^i^	1.957 (2)	N1—H1B	0.8900
Co2—N2^ii^	1.957 (2)	N1—H1C	0.8900
Co2—N2	1.957 (2)	N2—H2B	0.9101
Co2—N3^ii^	1.966 (2)	N2—H2A	0.9100
Co2—N3^i^	1.966 (2)	N2—H2	0.9100
Co2—N3	1.966 (2)	N3—H3B	0.9100
Co3—N1^iii^	1.965 (3)	N3—H3A	0.9099
Co3—N1^iv^	1.965 (3)	N3—H3	0.9100
Co3—N1^v^	1.965 (3)	C1—C2^ii^	1.553 (4)
Co3—N1	1.965 (3)	C2—C1^i^	1.553 (4)
Co3—N1^vi^	1.965 (3)	O1W—H1WA	0.8700
Co3—N1^vii^	1.965 (3)	O1W—H1W	0.8700
Cl1—Cl1'^viii^	1.041 (17)	O2W—H2WA	0.8699
Cl1—Cl1'	1.041 (17)	O2W—H2W	0.8700
			
O3^i^—Co1—O3^ii^	90.71 (7)	N1^iv^—Co3—N1^vi^	91.36 (15)
O3^i^—Co1—O3	90.71 (7)	N1^v^—Co3—N1^vi^	180.0 (2)
O3^ii^—Co1—O3	90.71 (7)	N1—Co3—N1^vi^	88.64 (15)
O3^i^—Co1—O4^ii^	78.45 (7)	N1^iii^—Co3—N1^vii^	91.36 (15)
O3^ii^—Co1—O4^ii^	104.20 (7)	N1^iv^—Co3—N1^vii^	88.64 (15)
O3—Co1—O4^ii^	161.53 (7)	N1^v^—Co3—N1^vii^	88.64 (15)
O3^i^—Co1—O4^i^	104.20 (7)	N1—Co3—N1^vii^	180.0 (2)
O3^ii^—Co1—O4^i^	161.53 (7)	N1^vi^—Co3—N1^vii^	91.36 (15)
O3—Co1—O4^i^	78.45 (7)	Cl1'^viii^—Cl1—Cl1'	180.000 (2)
O4^ii^—Co1—O4^i^	89.66 (7)	C2—O3—Co1	115.64 (17)
O3^i^—Co1—O4	161.53 (7)	C1—O4—Co1	114.93 (16)
O3^ii^—Co1—O4	78.45 (7)	Co3—N1—H1A	109.5
O3—Co1—O4	104.20 (7)	Co3—N1—H1B	109.5
O4^ii^—Co1—O4	89.66 (7)	H1A—N1—H1B	109.5
O4^i^—Co1—O4	89.66 (7)	Co3—N1—H1C	109.5
N2^i^—Co2—N2^ii^	90.56 (10)	H1A—N1—H1C	109.5
N2^i^—Co2—N2	90.56 (10)	H1B—N1—H1C	109.5
N2^ii^—Co2—N2	90.56 (10)	Co2—N2—H2B	111.0
N2^i^—Co2—N3^ii^	91.47 (10)	Co2—N2—H2A	111.2
N2^ii^—Co2—N3^ii^	87.31 (10)	H2B—N2—H2A	102.8
N2—Co2—N3^ii^	177.07 (9)	Co2—N2—H2	112.7
N2^i^—Co2—N3^i^	87.31 (10)	H2B—N2—H2	109.7
N2^ii^—Co2—N3^i^	177.07 (9)	H2A—N2—H2	108.8
N2—Co2—N3^i^	91.47 (10)	Co2—N3—H3B	113.7
N3^ii^—Co2—N3^i^	90.74 (9)	Co2—N3—H3A	108.2
N2^i^—Co2—N3	177.07 (9)	H3B—N3—H3A	104.8
N2^ii^—Co2—N3	91.47 (10)	Co2—N3—H3	111.6
N2—Co2—N3	87.31 (10)	H3B—N3—H3	108.4
N3^ii^—Co2—N3	90.74 (9)	H3A—N3—H3	109.9
N3^i^—Co2—N3	90.74 (9)	O1—C1—O4	125.1 (3)
N1^iii^—Co3—N1^iv^	180.0 (2)	O1—C1—C2^ii^	119.5 (2)
N1^iii^—Co3—N1^v^	91.36 (15)	O4—C1—C2^ii^	115.4 (2)
N1^iv^—Co3—N1^v^	88.64 (15)	O2—C2—O3	125.7 (3)
N1^iii^—Co3—N1	88.64 (15)	O2—C2—C1^i^	118.7 (2)
N1^iv^—Co3—N1	91.36 (15)	O3—C2—C1^i^	115.5 (2)
N1^v^—Co3—N1	91.36 (15)	H1WA—O1W—H1W	108.3
N1^iii^—Co3—N1^vi^	88.64 (15)	H2WA—O2W—H2W	107.4

Symmetry codes: (i) −*x*+*y*+1, −*x*+1, *z*; (ii) −*y*+1, *x*−*y*, *z*; (iii) −*x*+*y*, −*x*, *z*; (iv) *x*−*y*, *x*, −*z*; (v) *y*, −*x*+*y*, −*z*; (vi) −*y*, *x*−*y*, *z*; (vii) −*x*, −*y*, −*z*; (viii) −*x*, −*y*, −*z*+1.

### Hydrogen-bond geometry (Å, º)

**Table:**

*D*—H···*A*	*D*—H	H···*A*	*D*···*A*	*D*—H···*A*
N1—H1*A*···Cl1′	0.89	2.62	3.176 (15)	121
N1—H1*A*···O1^iii^	0.89	2.27	3.055 (4)	147
N1—H1*B*···O2^ix^	0.89	2.40	2.950 (4)	120
N1—H1*B*···O2^x^	0.89	2.52	3.151 (4)	128
N2—H2···O4^i^	0.91	2.09	2.993 (3)	171
N2—H2*A*···O1*W*	0.91	2.18	3.047 (4)	160
N2—H2*B*···O2*W*	0.91	2.15	2.958 (3)	147
N3—H3···O3^xi^	0.91	2.09	2.988 (3)	172
N3—H3*A*···O2*W*	0.91	2.19	3.051 (3)	157
N3—H3*B*···O1*W*	0.91	2.36	3.120 (4)	141
O1*W*—H1*W*···O1	0.87	2.13	2.971 (4)	162
O1*W*—H1*WA*···O1^xii^	0.87	2.32	2.973 (4)	132
O2*W*—H2*W*···O2^xiii^	0.87	1.97	2.830 (3)	171
O2*W*—H2*WA*···O4^xiv^	0.87	2.06	2.868 (3)	154

Symmetry codes: (i) −*x*+*y*+1, −*x*+1, *z*; (iii) −*x*+*y*, −*x*, *z*; (ix) *y*−1, −*x*+*y*, −*z*; (x) *x*−1, *y*−1, *z*; (xi) −*y*+1, *x*−*y*, *z*+1; (xii) *x*−*y*, *x*, −*z*+1; (xiii) *x*, *y*, *z*+1; (xiv) −*x*+1, −*y*+1, −*z*+1.
